# Fluid overload and mortality in critically ill patients with severe heart failure and cardiogenic shock–An observational cohort study

**DOI:** 10.3389/fmed.2022.1040055

**Published:** 2022-11-17

**Authors:** Jan Waskowski, Matthias C. Michel, Richard Steffen, Anna S. Messmer, Carmen A. Pfortmueller

**Affiliations:** Department of Intensive Care Medicine, Inselspital, Bern University Hospital, University of Bern, Bern, Switzerland

**Keywords:** fluid overload, mortality, cardiogenic shock, heart failure, cardiac surgery, critically ill, ICU

## Abstract

**Objective:**

Patients with heart failure (HF) and cardiogenic shock are especially prone to the negative effects of fluid overload (FO); however, fluid resuscitation in respective patients is sometimes necessary resulting in FO. We aimed to study the association of FO at ICU discharge with 30-day mortality in patients admitted to the ICU due to severe heart failure and/or cardiogenic shock.

**Methods:**

Retrospective, single-center cohort study. Patients with admission diagnoses of severe HF and/or cardiogenic shock were eligible. The following exclusion criteria were applied: (I) patients younger than 16 years, (II) patients admitted to our intermediate care unit, and (III) patients with incomplete data to determine FO at ICU discharge. We used a cumulative weight-adjusted definition of fluid balance and defined more than 5% as FO. The data were analyzed by univariate and adjusted univariate logistic regression.

**Results:**

We included 2,158 patients in our analysis. 185 patients (8.6%) were fluid overloaded at ICU discharge. The mean FO in the FO group was 7.2% [interquartile range (IQR) 5.8–10%]. In patients with FO at ICU discharge, 30-day mortality was 22.7% compared to 11.7% in non-FO patients (*p* < 0.001). In adjusted univariate logistic regression, we did not observe any association of FO at discharge with 30-day mortality [odds ratio (OR) 1.48; 95% confidence interval (CI) 0.81–2.71, *p* = 0.2]. No association between FO and 30-day mortality was found in the subgroups with HF only or cardiogenic shock (all *p* > 0.05). Baseline lactate (adjusted OR 1.27; 95% CI 1.13–1.42; *p* < 0.001) and cardiac surgery at admission (adjusted OR 1.94; 95% CI 1.0–3.76; *p* = 0.05) were the main associated factors with FO at ICU discharge.

**Conclusion:**

In patients admitted to the ICU due to severe HF and/or cardiogenic shock, FO at ICU discharge seems not to be associated with 30-day mortality.

## Introduction

In recent years, positive fluid balance (FB) and fluid overload (FO) came into focus as side effects of fluid resuscitation (1, 2). FO is not considered a clinical condition but is rather a complication of fluid resuscitation and organ dysfunction (3). However, its occurrence has a significant impact on patient management and critical care outcomes (1).

Heart failure (HF) is one of the main cardiac diseases leading to intensive care unit (ICU) admission (18.6%) (4). Organ congestion is a classical feature of HF (4–7) that is caused by excess fluid or fluid redistribution into the extravascular space (7). In addition, during and after cardiac surgery (CS) or cardiac interventions, the infusion of considerable amounts of IV fluids may be necessary (7, 8) due to cardiopulmonary bypass (CPB) induced inflammation (8), blood loss, myocardial depression, rhythm disturbances, and impaired vascular tone (9). Therefore, additional iatrogenic FO is common at ICU admission in patients with HF and may affect critical care outcomes (10, 11).

Further, patients with heart failure often suffer from extravascular over-hydration due to fluid re-distribution while actually being intravascular fluid depleted (7). This may lead to the administration of further fluids for resuscitation purposes (5, 12–14) and thus further aggravates organ dysfunction (heart, lung, and kidneys), leading to a vicious circle of organ failure (7).

Some studies indicate that FO may be associated with increased mortality in critically ill adults admitted for cardiogenic reasons to the ICU (1, 15, 16) as well as critically ill children and children after heart surgery (11, 17). Only few studies (18–20) assessed FO as an independent risk factor for poor outcomes in patients with heart disease treated in the ICU. Moreover, these investigations are mainly restricted to either very specific subgroups of patients such as HF patients with sepsis (20) or patients on extracorporeal membrane oxygenation (ECMO) (14, 19), or to only peri-operative outcomes (20) and early FO (14, 19). Therefore, the aim of this cohort trial was to evaluate the association of FO with 30-day mortality in patients admitted to the ICU with severe HF and/or cardiogenic shock. Further, we also aimed to identify risk factors for FO in respective patients.

## Materials and methods

### Study set and ethical consideration

We conducted a single-center, retrospective cohort study at the Inselspital, University Hospital of Bern, Switzerland. Our ICU is a mixed medico-surgical ICU and the only provider of intensive care in this tertiary care center. We analyzed patient data from electronic patient charts of patients admitted to our ICU from 1st January 2014 to 30th June 2018.

### Ethical considerations

The competent ethics committee of the Canton of Bern (Kantonale Ethikkommission Bern), Switzerland, approved the study (BASEC no. 2018-00436). The individual informed consent was waived due to the retrospective character of the study and an approved general consent procedure. We conducted the study in accordance with the Declaration of Helsinki.

### Study population

**FIGURE 1:**
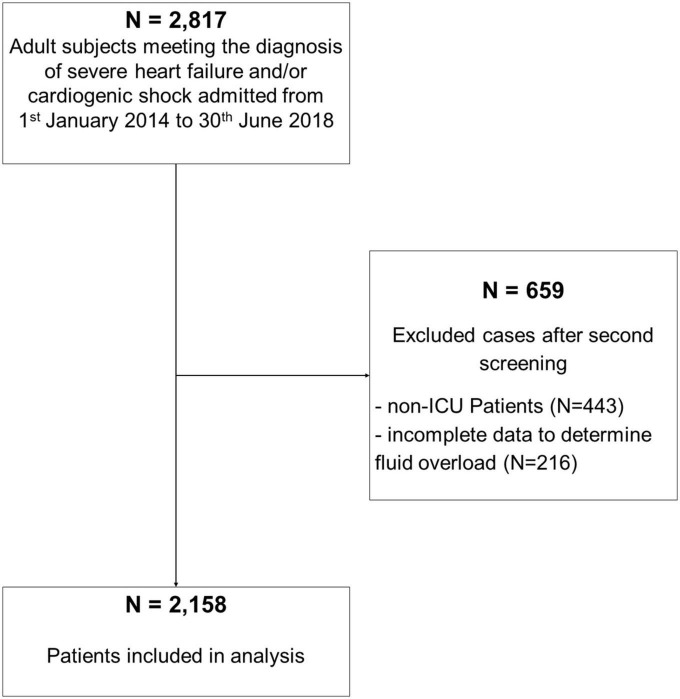
Strobe flowchart. ICU, intensive care unit.

We included adult ICU patients admitted during the study period with cardiogenic shock and/or severe heart insufficiency. Exclusion criteria were as follows: (i) patients younger <16 years, (ii) insufficient data to calculate the percentage of FO (missing body weight, fluid data) at ICU discharge, and (iii) patients admitted to our intermediate care unit (IMC) (Figure 1 STROBE flowchart).

### Data extraction

This project is part of a large database on fluids, fluid overload, and electrolyte disorders in the critically ill (21). Insel Data Coordination Lab (IDCL) provided data for this project. The ICDL extracted the data from our hospital’s electronic medical databases (SAP ERP 6.07/Inselspital Bern © SAP Schweiz 2018, Centricity Critical Care 8.1 © GE Electric Company, Boston, MA, USA, 2018, Xserv.4 R19.3 © ixmid GmbH, Köln, Germany, 2020, ipdos V7.16, © CompuGroup Medical Schweiz AG, Bern, Switzerland). We identified eligible patients through a search in the hospital’s administrative electronic database (SAP). Following variables on included patients were extracted: demographic data (e.g., age, sex), diagnoses and comorbidities, admission data including body weight (i.e., for surgical patients measured pre-operative body weight), reason(s) for admission, and need for mechanical ventilation, vasopressors, mechanical cardiovascular support, surgical or interventional procedures, as well as laboratory findings at ICU admission. Additionally, outcome variables, (mortality, LOS, need for renal replacement therapy, mechanical cardiovascular support on ICU, surgery or interventions, and infections while on ICU) we extracted. We calculated the percentage of FO at ICU discharge using the formula given under definitions (16). Diagnosis and underlying diseases were based on the International Statistical Classification of Diseases and Related Health Problems, 10th revision (ICD-10) (22). We extracted the mortality data from the Swiss National Death Registry (ZAS, Zentrales Sterberegister).

### Objectives

The primary objective of this retrospective cohort trial was the impact of FO at ICU discharge on 30-day mortality in critically ill patients with cardiogenic shock and/or severe heart failure at ICU admission. The secondary aim was to identify risk factors for FO in patients with HF.

### Definitions

#### Fluid overload

Fluid overload (FO) was defined as a weight-adjusted cumulative FB (= total fluid in–total fluid out) at ICU discharge ≥5% (23, 24). It was estimated using the following formula (16, 24, 25):


% FB=(total fluid in−total fluid out)/admission body weight×100.

#### Severe heart failure

The definition of severe HF is based on the New York Heart Association (NYHA) functional classification. Severe HF includes patients with marked limitation of activity (class III) or symptoms at rest (class IV) (26). For this study, all patients on the ICU admitted with severe heart failure as per the APACHE diagnosis were investigated.

#### Cardiogenic shock

Cardiogenic shock was defined by inadequate cardiac output due to primary cardiac dysfunction, leading to tissue hypoperfusion and organ failure (26). For this study, all patients on the ICU admitted with cardiogenic shock as per the APACHE diagnosis were investigated.

### Statistical analysis

We performed all statistical analyses using the software SPSS for Windows (version 25; SPSS Inc., Chicago, IL, USA). We tested for normal distribution using the Kolmorgorov–Smirnov test or the Pearson’s chi-squared test. Student’s *t*-test for normally distributed data and the Mann–Whitney U test for non-normally distributed data were used to compare quantitative variables between groups. Chi square test or Fisher’s exact test were used for qualitative variables, as appropriate. Statistical significance was assumed for *p* < 0.05. For the following variables, we identified more than 5% missing values: APACHE, baseline creatinine, baseline bicarbonate, baseline base excess (BE), and baseline lactate. We applied multiple imputations (*n* = 10) for respective variables (see Supplementary Figure 1 for imputation models). Univariate binominal followed by adjusted univariate multinomial regression models with stepwise inclusion of relevant clinical variables for 30-day mortality were used to assess the relationship of FO with 30-day mortality. To identify relevant risk factors for FO, we applied univariate followed by multivariate logistic regression. We identified relevant confounders for 30-day mortality and FO at ICU discharge by using univariate and multivariate Cox regression analysis. Variables with statistically significant differences between the outcome groups (*p* ≤ 0.05) were then used for adjustment of univariate multinomial regression analysis. A survival curve was constructed using confounder adjusted Cox regression models for patients with/without FO at ICU discharge.

## Results

### Baseline characterization of study cohort

Out of 2,817 patients, meeting the diagnosis of severe HF and/or cardiogenic shock at admission, 2,158 patients were included in our analysis (Figure 1). Among the cohort, 185 (8.6%) patients had FO at ICU discharge. The mean FO in the FO group was 7.2% [interquartile range (IQR) 5.8–10%] corresponding to a mean positive fluid balance of 5,224 ml (IQR 4,271–7,678 ml) compared to a mean 0% or 4 ml (IQR −1.5–1.2% and −1,224–991 ml, respectively) in the no FO group. Patient demographics are shown in Table 1.

**TABLE 1:** Patient demographics.

	Total	FO	no FO	*P-value*
		
	*N* = 2158	*N* = 185	*N* = 1973	
***Demographics***				
Sex, male (%)	1,476 (68.4)	121 (65.4)	1,355 (68.7)	0.36
Age [years], median (IQR)	68 (59–76)	71 (64–78)	68 (59–76)	**0.001**
Type of admission				** < 0.0001**
Planned, *n* (%)	766 (35.5)	100 (54.1)	666 (33.8)	
Emergency, *n* (%)	1,392 (64.5)	85 (45.9)	1,307 (66.2)	
Prior chronic kidney disease, *n* (%)	964 (44.7)	79 (42.7)	885 (44.9)	0.59
Chronic dialysis, *n* (%)	78 (3.6)	6 (3.2)	72 (3.6)	1
Chronic liver disease, *n* (%)	193 (8.9)	26 (14.1)	167 (8.5)	**0.02**
Cancer, *n* (%)	176 (8.2)	25 (13.5)	151 (7.7)	**0.01**
Prior respiratory disease, *n* (%)	920 (60.9)	64 (53.3)	856 (61.6)	0.08
***Clinical presentation at admission to ICU***				
APACHE II, mean (SD)*	20.5 (10.1)	23.2 (11)	20.3 (10)	**<0.001**
Cardiogenic shock, *n* (%)	709 (32.9)	67 (36.2)	642 (32.5)	0.33
Severe heart failure, *n* (%)	1,602 (74.2)	130 (70.3)	1,472 (74.6)	0.22
Cardiac surgery at admission, *n* (%)	1,048 (48.6)	118 (63.8)	930 (47.1)	**<0.001**
Cardiac intervention at admission, *n* (%)	144 (6.7)	15 (8.1)	129 (6.5)	0.44
Need for mechanical cardiovascular assistance, *n* (%)	90 (4.2)	15 (8.1)	75 (3.8)	**0.01**
Need for mechanical ventilation, *n* (%)	1,124 (52.3)	121 (65.4)	1,003 (51)	**<0.001**
Need for vasoactives, *n* (%)	109 (5.1)	10 (5.4)	99 (5)	0.86
Infection at admission, *n* (%)	609 (28.2)	41 (22.2)	568 (28.8)	0.06
***Laboratory parameters at baseline***				
Baseline creatinine [μmol/l], mean (SD)*	129.4 (93.7)	123.3 (97)	130 (93.4)	0.36
Baseline bicarbonate [mmol/l], mean (SD)*	23.3 (4.3)	21.6 (3.7)	23.4 (4.3)	**<0.001**
Baseline lactate [mmol/l], mean (SD)*	2.3 (1.9)	3.7 (2.9)	2.2 (1.7)	**<0.001**
Baseline base excess [mmol/l], mean (SD)*	−1.0 (4)	−2.7 (4.3)	−0.9 (4.2)	**<0.001**
***ICU stay***				
Surgery or intervention during ICU stay, *n* (%)	378 (17.5)	56 (30.3)	322 (16.3)	**<0.001**
Infection during ICU stay, *n* (%)	363 (16.8)	39 (21.1)	324 (16.4)	0.1
Need for cardiac assist device during ICU stay, *n* (%)	177 (8.2)	23 (12.4)	154 (7.8)	**0.04**
ICU discharge balance [ml], median (IQR)	103 (−1,038–1,519)	5,224 (4,271–7,678)	4 (−1,224–991)	**<0.001**
AKI any KDIGO stage, *n* (%)	1,194 (55.3)	109 (58.9)	1,085 (55)	0.32
RRT during ICU stay, *n* (%)	186 (8.6)	21 (11.4)	165 (8.4)	0.17
Diuretics of any type in first 72 h, *n* (%)	1,087 (50.4)	92 (49.7)	995 (50.4)	0.88
Cumulative ICU furosemid equivalent first 72 h [mg], median (IQR)	0 (0–40)	0 (0–20)	0 (0–40)	0.25
LOS ICU [days], median (IQR)	0.99 (0.78; 2.48)	1.81 (0.9; 3.27)	0.96 (0.76; 2.35)	**<0.001**

Medians and interquartile range (IQR) or mean and standard deviation (SD) or total numbers (relative frequencies) are given. Student’s *t*-test (normally distributed data) or the Mann–Whitney U test (MW) for non-normally distributed data were used. *P*-values were obtained by Chi-squared test or Fisher’s exact test (F) (where appropriate). APACHE II, Acute Physiology and Chronic Health Evaluation II score; ICU, intensive care unit; AKI, acute kidney injury; KDIGO, Kidney Disease: Improving Global Outcomes; RRT, renal replacement therapy; LOS, length of stay. *Combined data after 10 imputations. Bold values represent the *p* < 0.05.

### Identifying risk factors for 30-day mortality

A univariate followed by a multivariate Cox regression analysis was performed to identify risk factors for 30-day mortality in this patient cohort. Age, chronic liver disease, cancer, prior respiratory disease, cardiac surgery at admission, baseline creatinine, baseline bicarbonate, and baseline lactate, as well as the need for a cardiac assist device after ICU admission, were all independently associated with 30-day mortality (all *p* ≤ 0.05) (Supplementary Table 1).

### Identifying risk factors associated with fluid overload at discharge

Univariate followed by multivariate binary logistic regression identified age, chronic liver disease, cancer, cardiac surgery at admission, baseline lactate, and any surgery or intervention during the ICU stay as risk factors for FO at ICU discharge (all *p* ≤ 0.05) (Table 2). Emergency admission was inversely associated with FO at ICU discharge (OR 0.41; 95% CI 0.23–0.74, *p* = 0.003) (Table 2).

**TABLE 2:** Univariate and multivariate logistic regression analysis on risk factors for fluid overload at discharge.

	FO	no FO	Univariate model (unadjusted)		Multivariate model (adjusted)	
	*N* = 185	*N* = 1973	OR (95% CI)	*P-value*	OR (95% CI)	*P-value*
***Demographics***						
Sex, [male], *n* (%)	121 (65.4)	1,355 (68.7)	0.86 (0.63–1.19)	0.36		
Age [years], median (IQR)	71 (64–78)	68 (59–76)	1.02 (1.01–1.02)	**<0.001**	1.05 (1.03–1.07)	**<0.001**
Type of admission, emergency, *n* (%)	85 (45.9)	1,307 (66.2)	0.43 (0.32–0.59)	**<0.001**	0.41 (0.23–0.74)	**0.003**
Prior chronic kidney disease, *n* (%)	79 (42.7)	885 (44.9)	0.92 (0.84–1.004)	0.06		
Chronic dialysis, *n* (%)	6 (3.2)	72 (3.6)	0.89 (0.69–1.14)	0.38		
Chronic liver disease, *n* (%)	26 (14.1)	167 (8.5)	1.77 (1.55–2.02)	**<0.001**	2.48 (1.37–4.46)	**0.003**
Cancer, *n* (%)	25 (13.5)	151 (7.7)	1.89 (1.65–2.12)	**<0.001**	2.13 (1.12–4.06)	**0.02**
Prior respiratory disease, *n* (%)	64 (53.3)	856 (61.6)	0.71 (0.62–0.8)	**<0.001**	0.93 (0.6–1.42)	0.72
***Clinical presentation at admission to ICU***						
APACHE II, mean (SD)*	23.2 (11)	20.3 (10)	1.03 (1.01–1.05)	**<0.001**	1.0 (0.98–1.03)	0.79
Cardiogenic shock, *n* (%)	67 (36.2)	642 (32.5)	1.18 (1.07–1.29)	**<0.001**	0.94 (0.59–1.5)	0.78
Severe heart failure, *n* (%)	130 (70.3)	1,472 (74.6)	0.8 (0.73–0.89)	**<0.001**	0.92 (0.55–1.55)	0.77
Cardiac Surgery at admission, *n* (%)	105 (56.8)	712 (36.1)	2.33 (1.71–3.15)	**<0.001**	1.94 (1.0–3.76)	**0.05**
Cardiac intervention, *n* (%)	15 (8.1)	129 (6.5)	1.26 (1.07–1.49)	**0.007**	2.03 (0.91–4.52)	0.08
Need for mechanical cardiovascular assistance, *n* (%)	15 (8.1)	75 (3.8)	2.23 (1.87–2.65)	**<0.001**	0.97 (0.3–3.11)	0.96
Need for mechanical ventilation, *n* (%)	121 (65.4)	1,003 (51)	1.82 (1.65–2)	**<0.001**	0.92 (0.57–1.51)	0.75
Need for vasoactives, *n* (%)	10 (5.4)	99 (5)	1.08 (0.88–1.32)	0.46		
Infection at admission, *n* (%)	41 (22.2)	568 (28.8)	0.7 (0.63–0.79)	**<0.001**	0.88 (0.5–1.53)	0.64
***Laboratory parameters at baseline***						
Baseline creatinine [μmol/l], mean (SD)*	123.3 (97)	130 (93.4)	1	0.35		
Baseline bicarbonate [mmol/l], mean (SD)*	21.6 (3.7)	23.4 (4.3)	0.89 (0.86–0.93)	**<0.001**	0.85 (0.68–1.07)	0.17
Baseline lactate [mmol/l], mean (SD)*	3.7 (2.9)	2.2 (1.7)	1.31 (1.23–1.39)	**<0.001**	1.27 (1.13–1.42)	**<0.001**
Baseline base excess [mmol/l], mean (SD)*	−2.7 (4.3)	−0.9 (4.2)	0.9 (0.87–0.94)	**0.02**	1.1 (0.88–1.36)	0.4
***ICU stay***						
Surgery or intervention during ICU stay, *n* (%)	56 (30.3)	322 (16.3)	2.23 (2.01–2.46)	**<0.001**	1.76 (1.03–2.99)	**0.04**
Infection during ICU stay, *n* (%)	39 (21.1)	324 (16.4)	1.36 (1.22–1.52)	**<0.001**	1.52 (0.84–2.77)	0.17
Need for cardiac assist device during ICU stay, *n* (%)	23 (12.4)	154 (7.8)	1.68 (1.46–1.93)	**<0.001**	1.31 (0.47–3.65)	0.6

APACHE II, Acute Physiology and Chronic Health Evaluation II score; ICU, Intensive care unit; FO, Fluid overload. *Combined data after 10 imputations. Bold values represent the *p* < 0.05.

### Association of fluid overload with 30-day mortality

In patients with FO at ICU discharge, we observed a 30-day mortality of 22.7% (*n* = 42) compared to 11.7% (*n* = 231) in patients without FO at ICU discharge. In the unadjusted regression analysis, using FO > 5% at ICU discharge as a categorical variable, FO was associated with 30-day mortality [OR 2.22; 95% Confidence Interval (CI) 1.53–3.21, *p* < 0.001]. When applying FO as a continuous variable, unadjusted univariate regression analysis shows no association with 30-day mortality in our cohort (OR 1; 95% CI 0.99–1.01).

After adjustment for the previously identified risk factors for 30-day mortality and fluid overload, we observed no association of FO at discharge with 30-day mortality in patients admitted to the ICU with severe HF and/or cardiogenic shock in univariate multinomial regression analysis (OR 1.48; 95% CI 0.81–2.71, *p* = 0.2).

**FIGURE 2:**
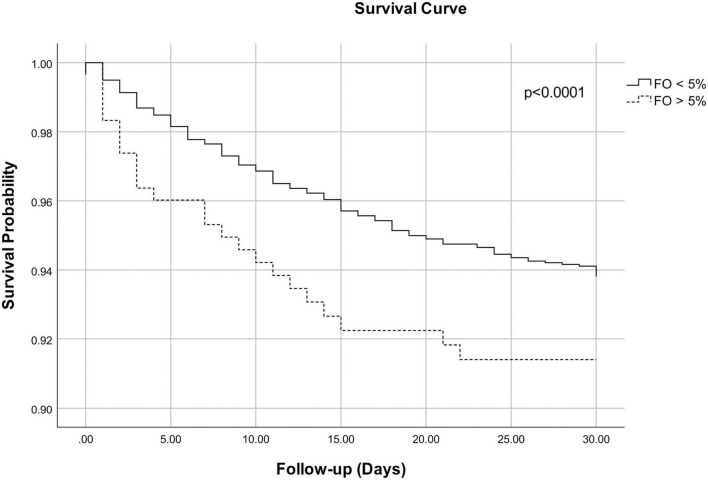
Adjusted Cox regression survival curves for 30-day mortality for patients with fluid overload <5% and fluid overload >5% at intensive care unit (ICU) discharge. The curves are adjusted for age, chronic liver disease, cancer, prior respiratory disease, cardiac surgery at admission, baseline creatinine, baseline bicarbonate, baseline lactate, and the need for a cardiac assist device after ICU admission. Log likelihood test *p* < 0.0001.

Adjusted Cox regression survival curves showed a small but statistical significant difference for cumulative survival at 30 days between groups (log likelihood test *p* < 0.0001) (Figure 2).

### Subgroup analysis

Subgroup analysis for patients admitted with severe HF and cardiogenic shock at admission showed similar results for the primary endpoint (all *p* > 0.5) (Supplementary Tables 2A,B).

When comparing subgroups (FO < 5%, 5–10%, >10%), only the FO > 10% group remained an independent predictor for 30d mortality after adjustment [3.61 (1.39–9.37, *p* = 0.008)] (Supplementary Tables 3A–C).

## Discussion

In our observational cohort study, we did not observe an association of FO at ICU discharge with 30-day mortality after adjustment with Cox-regression analysis identified confounders in patients admitted to the ICU with severe HF and/or cardiogenic shock. Important factors contributing to fluid overload in this patient collective were elevated baseline lactate, cardiac surgery at admissions, and prior liver disease.

In our study, we were not able to show an independent association between FO at ICU discharge and mortality in critically ill patients with cardiogenic shock/severe heart failure. This contrasts with previous studies in subgroups of cardiac patients admitted to the ICU, e.g., with HF and sepsis, after cardiac surgery (18, 20, 27). There are several reasons why our results differ from previous investigations.

First, compared to some of those studies (18, 27), we used a cumulative weight-adjusted definition of fluid balance and defined more than 5% as FO. Currently, there are various definitions used in the literature to describe and examine fluid accumulation in the critically ill patient. The most common definition uses a positive FB as a surrogate for fluid accumulation on a daily or cumulative basis (18, 27, 28). However, nurse-registered FB was shown to be inaccurate (29) and the association of daily weight gain with FB is poor (29–31). To improve accuracy, pediatric intensive care physicians suggested a weight-adjusted approach to FB, which was adopted by adult intensive care (24, 28) and was used in this investigation (25, 32). These approaches better account for the patients’ baseline total body water with the surrogate of body weight and improve comparability between patients (24, 32). FO of 5% or higher was shown to be inversely associated with survival in critical illness (1, 23). Nevertheless, as FB is part of the FO formula, the same limitations arise. Namely, besides arithmetical inaccuracy and lack of data (29), FB does not account for insensible fluid losses (30) and volume losses, e.g., due to bleeding before ICU admission (23). Moreover, in some ICU patients, it may be difficult to determine the patients’ true baseline body weight. The difference regarding the impact of FO on ICU survival in patients with severe HF and/or cardiogenic shock between our trial and others might be explained by the choice of definitions in the respective trials. This is supported by a recent investigation comparing the fluid accumulation index (FAI) to FB. The FAI consists of the ratio of FB to fluid intake as a measure of the patients’ ability to regulate his body water (33). A retrospective cohort study in HF patients with septic shock demonstrated an association of the FAI with mortality, while there was no association with FB (20). Moreover, after adjusting for confounders, some could show that early positive weight-adjusted FB or FAI remains an independent factor for mortality in patients with overt fluid accumulation (14, 19, 20), while others could not demonstrate an association of early positive or negative FB with the incidence of AKI and mortality (18, 27).

Second, in our study, we examined the association of FO at ICU discharge, while previous studies mostly focused on perioperative FO (12 h) (27), as well as early FB (within 2 days) (18–20). The optimal time point of FO assessment (or FB as a surrogate marker) is not yet clarified. The use of very early FB or FO to estimate survival in patients with HF/cardiogenic shock may be problematic for several reasons. Early critical illness may demand fluid administration for resuscitation and may reflect disease severity (28). This may also be true in patients with congestive HF (12, 34). Further, in patients with pre-existing severe HF and/or cardiogenic shock, recent guidelines (26) and reviews (12) underline that decongestion is the cornerstone of therapy and fluid resuscitation is restricted to specific situations such as isolated right heart failure (26) or sepsis (12). Together with the growing recent evidence on the adverse effects of FO (1), treating physicians seem to be increasingly sensibilised to the potential detrimental effects of fluid administration in those patients and restrain from unnecessary fluid administration. This may explain the low incidence of FO at discharge in our cohort (only 8.6%) in comparison to the significantly higher FO rate in other studies that used an earlier assessment point (18).

Third, in contrast to our investigation, previous studies on fluid accumulation in cardiac patients mostly chose short-term outcomes such as duration of mechanical ventilation (27), post-operative mortality (18), or in-hospital mortality (20). The longer observation period in our study might explain why we did not find an independent association between FO and 30-day mortality. Overall mortality in our population (data not shown) was comparable to previous published data on the 30-day mortality of patients with severe HF (35) but was slightly higher than mortality observed in perioperative studies in cardiac surgery (18, 27, 36).

In our retrospective study, we observed that patients with HF or cardiogenic shock, cardiac surgery at admission, and baseline lactate are associated with FO at ICU discharge. Disease severity or pre-existing renal disease are not associated with FO at discharge. This is consistent with a recent analysis of our group on risk factors for FO in more than 14,000 patients in the general ICU (21). Elevated lactate or impaired lactate clearance are well-described risk factors for mortality in the critically ill with cardiac disease (37–39) and are regarded as surrogate for microcirculatory failure (40). Recent guidelines recommend the measurement of lactate in patients with cardiogenic shock (13, 26). However, recommendations for the management of patients with sepsis differ significantly from those for the treatment of patients with severe HF and cardiogenic shock regarding fluid management (fluid administration vs. decongestion) (13, 26). Notably, there are some subgroups of HF patients (peri-surgical, sepsis, and right ventricular failure) that may necessitate fluid resuscitation to some extent (12, 26), but fluid management of those patients remains particularly challenging and should be tailored to individual needs. As outlined above, guidance and high-quality evidence are currently lacking, and the role of fluid resuscitation in HF patients needs further clarification in RCTs.

### Limitations

Our study is of a retrospective, monocentric, and explorative study design, and therefore all inherent limitations that are driven by this study design apply, such as for, i.e., the reason for fluid administration is unknown. Second, we did not account for peri-interventional and peri-surgical fluid in/or outputs. Therefore, total FO at ICU discharge and the percentage of patients with FO may be underestimated and may contribute to the low incidence of FO in our cohort. To overcome this limitation, we used the measured pre-operative weight available as the baseline weight for the calculation of FO. Further, as previously mentioned, incomplete data as well as incorrect measurements and recording of fluid data at beside and during data collection may flaw calculations of fluid balance. Moreover, we did not account for insensible fluid losses (diarrhea, perspiration) in our data. Fifth, we did not account and adjust intra- and extravascular fluid status at ICU admission. However, the recognition of such is essential for correctly treating patients with severe HF/cardiogenic shock. Sixth, as outlined above, there is no consensus in the literature on the definition of fluid overload, and current definitions all have their drawback. While fluid accumulation is in reality a continuous process, fluid overload implies a dichotomous relationship. Currently, it is unclear what the adequate cut-off for determination of outcome is respectively beyond what percentage FO becomes relevant for patient outcomes. In this investigation, we applied a cut-off value of 5% or more in accordance with current literature (23, 24). However, we cannot exclude that the use of a continuous scale or a different cut-off would have led to different results. Moreover, we did not examine the impact of an overly negative FB on mortality in this patient cohort. Some data suggest that also a negative FB may negatively influence long-term mortality (24). Our adjusted Cox-regression analysis showed a significant relationship between FO and 30-day mortality, which was not present in the primary analysis. As the set of confounders for statistical adjustment was the same for both analyses, we assume that other important unaccounted confounders such as, i.e., fluid received after ICU discharge, diuretics received, or subgroups of patients, e.g., after cardiac surgery, may have significantly influenced this finding. Unfortunately, due to the retrospective nature of this trial and the limited number of patients and events in our cohort, we do not have the means to further investigate this important finding. Additionally, even though, the number of the patients is high (about 2,000, about 300 died), the group of patients is very in-equilibrate. Therefore, type 2 error remains a possibility.

## Conclusion

In our observational cohort study, we did not observe an association of FO at ICU discharge with 30-day mortality after adjustment for typical confounders in patients admitted to the ICU with severe HF and/or cardiogenic shock. This contrasts to other investigations in this patient collective. However, current literature is inconsistent regarding FO definitions, time-point of assessment, and outcomes assessed, which hampers comparability between studies. In this study, important factors contributing to fluid overload were elevated baseline lactate, cardiac surgery at admission, and prior liver disease. Further high-quality investigations are needed.

## Data availability statement

The raw data supporting the conclusions of this article will be made available by the authors, without undue reservation.

## Ethics statement

The studies involving human participants were reviewed and approved by Kantonale Ethikkommission Bern. Written informed consent for participation was not required for this study in accordance with the national legislation and the institutional requirements.

## Author contributions

CP designed the study and supervised the conduct of the study and data collection. JW and MM performed the study assessments, data cleaning, data preparation, and statistical analysis. JW and CP drafted the manuscript. MM, AM, and RS revised the manuscript for important intellectual content. All authors read and approved the final draft.
