# Supplementary material for: Fluid overload and mortality in critically ill patients with severe heart failure and cardiogenic shock–An observational cohort study
Source: Front Med (Lausanne). 2022 Nov 17;9:1040055. doi: 10.3389/fmed.2022.1040055 (PMC9712448; doi:10.3389/fmed.2022.1040055)
Supplement: Supplementary file 1 [file Data_Sheet_1.docx]

Supplemental Digital Content

This appendix has been provided by the authors to provide additional information

Supplement to:

**Fluid overload and mortality in critically patients with severe heart failure – a retrospective cohort study**

Jan Waskowski, MD^1^, Matthias C. Michel, MD^1^, Richard Steffen MD^1^, Anna S. Messmer^1^ ,Carmen A. Pfortmueller, MD^1^

Corresponding Author:

Dr. med. Jan Waskowski

Dept. of Intensive Care Medicine

Inselspital, Bern University Hospital

Freiburgstrasse 18

CH-3010 Bern, Switzerland

[Jan.Waskowski@insel.ch](mailto:Jan.Waskowski@insel.ch)

+41-31-632-5300


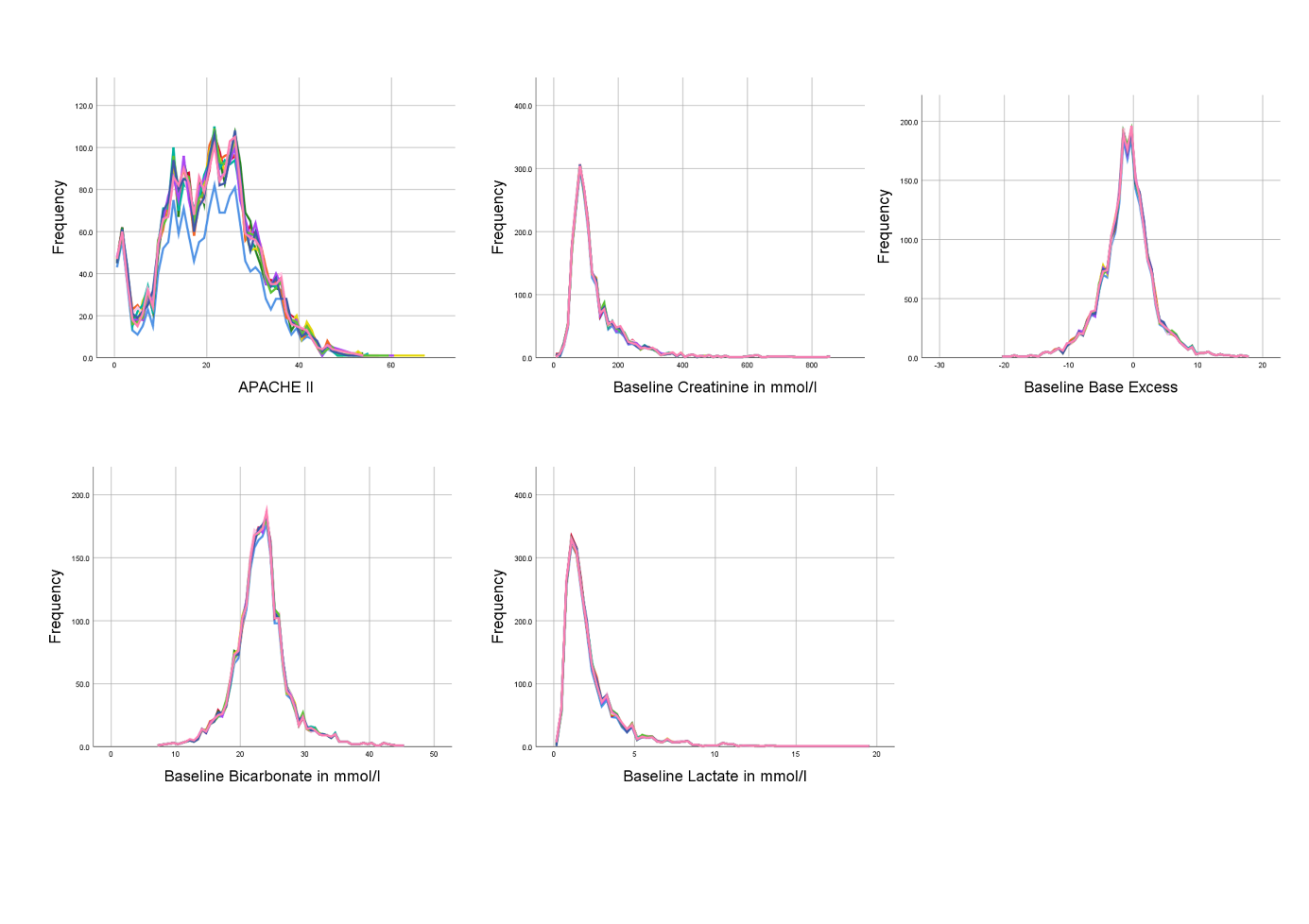


**Supplement Figure 1:** Explanatory variables on admission with missing values (%): APACHE IV score (23%), baseline creatinine (5.6%), baseline base excess (5.3%), baseline bicarbonate (5.3%), and baseline lactate (5.3%). Variables were imputed using multiple imputation (no. datasets = 10).

|  | **Univariate model** | | **Multivariate model** | |
| --- | --- | --- | --- | --- |
|  | HR (95% CI) | p-value | HR (95% CI) | p-value |
| *Demographics* | | | | |
| Age [years] | 1.03 (1.02 - 1.03) | **<0.001** | 1.04 (1.02 - 1.05) | **<0.001** |
| Type of admission (planned) | 3.41 (3.08 - 3.78) | **<0.001** | 0.98 (0.6 - 3.01) | 0.94 |
| Chronic liver disease | 2.87 (2.63-3.13) | **<0.001** | 2.14 (1.52 -3.01) | **<0.001** |
| Cancer | 2.65 (2.42 - 2.9) | **<0.001** | 1.62 (1.11 - 2.37) | **0.01** |
| Prior respiratory disease | 1.56 (1.42 - 1.71) | **<0.001** | 1.4 (1.0 - 1.96) | **0.05** |
| *Clinical presentation at admission to ICU* | | | | |
| APACHE II | 1.03 (1.02 - 1.04) | **<0.001** | 1.01 (0.99 - 1.02) | 0.48 |
| Cardiac Surgery at admission | 6.04(4.0 - 9.12) | **<0.001** | 6.41(3.24 - 12.71) | **<0.001** |
| Infection at admission | 2.28 (2.12 - 2.45) | **<0.001** | 1.32 (0.96 - 1.8) | 0.09 |
| Need for mechanical cardiovascular assistance | 1.24 (1.05 - 1.46) | **0.01** | 0.8 (0.33 - 1.85) | 0.57 |
| Need for mechanical ventilation | 0.58 (0.54 - 0.62) | **<0.001** | 1.1 (0.79 -1.51) | 0.58 |
| *Laboratory parameters at baseline* | | | | |
| Baseline creatinine, [µmol/l] | 1.003(1.002- 1.004) | **<0.001** | 1.0 (1.0 - 1.003) | **0.01** |
| Baseline bicarbonate, [mmol/l], | 0.96 (0.93 - 0.99) | **0.01** | 1.12(1.0 - 1.24) | **0.05** |
| Baseline lactate, [mmol/l] | 1.16 (1.12 - 1.21) | **<0.001** | 1.08(1.01 - 1.15) | **0.04** |
| Baseline base excess, [mmol/l] | 0.94 (0.92 - 0.97) | **<0.001** | 0.92(0.82 - 1.03) | 0.14 |
| *ICU stay* | | | | |
| Need for cardiac assist device during ICU stay | 1.43(1.28 - 1.61) | **<0.001** | 2.6 (1.36 - 5.0) | **0.004** |
| Infection during ICU stay | 1.99(1.83 - 2.14) | **<0.001** | 0.95(0.67 - 1.36) | 0.79 |
| Surgery or intervention during ICU stay | 1.47(1.35 - 1.6) | **<0.001** | 1.17(0.8 - 1.74) | 0.42 |
| Fluid overload >5% | 1.94(1.76-2.14) | **<0.001** | 1.38 (0.86 - 2.2) | 0.18 |

**Supplement Table 1:** Univariate and multivariate Cox Regression Analysis (30-days mortality). HR: Hazard rate; APACHE II: Acute Physiology and Chronic Health Evaluation II score; ICU: intensive care unit

| 1. **Heart failure** | **FO** | **no FO** | **Univariable model (unadjusted)** | | **Univariate model (adjusted)*** | |
| --- | --- | --- | --- | --- | --- | --- |
|  | N = 130 | N = 1472 | OR (95% CI) | p-value | OR (95% CI) | p-value |
| 30-day mortality, n (%) | 34 (26.2) | 210 (14.3) | 2.13 (1.4 - 3.23) | **0.001** | 1.84 (0.96 – 3.5) | 0.07 |
|  |  |  |  |  |  |  |
| 1. **Cardiogenic shock** | **FO** | **no FO** | **Univariate model (unadjusted)** | | **Univariate model (adjusted*)** | |
|  | N = 67 | N = 642 | OR (95% CI) | p-value | OR (95% CI) | p-value |
| 30-day mortality, n (%) | 17 (25.4) | 71 (11.1) | 2.73 (1.5 - 5) | **0.003** | 1.09 (0.43 - 2.79) | 0.85 |

**Supplement Table 2a-b:** Subgroup analysis according to type of cardiac disease.

*adjusted for age, chronic liver disease, cancer, prior respiratory disease, cardiac surgery at admission, baseline creatinine, baseline bicarbonate, baseline lactate, need for cardiac assist device after ICU admission.

|  | **FO < 5%** | **FO 5-10%** | **Univariable model (unadjusted)** | | **Univariate model (adjusted)*** | |
| --- | --- | --- | --- | --- | --- | --- |
|  | N = 1971 | N = 139 | OR (95% CI) | p-value | OR (95% CI) | p-value |
| 30-day mortality, n (%) | 231 (11.7) | 18 (12.9) | 1.12 (0.67-1.87) | **0.68** | - | - |
|  |  |  |  |  |  |  |
|  | **FO 5-10%** | **FO >10%** | **Univariate model (unadjusted)** | | **Univariate model (adjusted*)** | |
|  | N = 139 | N = 48 | OR (95% CI) | p-value | OR (95% CI) | p-value |
| 30-day mortality, n (%) | 18 (12.9) | 24 (50) | 6.72 (3.17-14.26) | **<0.001** | 2.43 (0.72 – 8.14) | 0.15 |
|  |  |  |  |  |  |  |
|  | **FO < 5%** | **FO >10%** | **Univariable model (unadjusted)** | | **Univariate model (adjusted*)** | |
|  | N = 1971 | N = 48 | OR (95% CI) | p-value | OR (95% CI) | p-value |
| 30-day mortality, n (%) | 231 (11.7) | 24 (50) | 7.53 (4.21-13.48) | **<0.001** | 3.61 (1.39-9.37) | **0.008** |

**Supplement Table 3 a-c:** Subgroup analysis according to amount of fluid overload (FO).

*adjusted for age, chronic liver disease, cancer, prior respiratory disease, cardiac surgery at admission, baseline creatinine, baseline bicarbonate, baseline lactate, need for cardiac assist device after ICU admission.
